# Renal replacement therapy and concurrent fluconazole therapy increase linezolid-related thrombocytopenia among adult patients

**DOI:** 10.1038/s41598-022-13874-y

**Published:** 2022-06-14

**Authors:** Yueh-Chun Hsu, Szu-Ying Chen, Yung-Jun Hung, Yu-Wei Huang

**Affiliations:** 1 Department of Pharmacy, Kaohsiung Chang Gung Memorial Hospital, Kaohsiung, 833 Taiwan; 2 Department of Anesthesiology, Emergency and Critical Care Center, E-Da hospital, I-Shou University, Kaohsiung, 824 Taiwan; 3 Department of Nursing, Fooyin University, Kaohsiung, 831 Taiwan; 4 Division of Occupational Medicine, E-Da Hospital, I-Shou University, Kaohsiung, 824 Taiwan; 5 Division of Surgical Intensive Care, Department of Critical Care Medicine, E-Da Hospital, I-Shou University, Kaohsiung, 824 Taiwan; 6 Department of Pharmacy, E-Da hospital, Kaohsiung, 824 Taiwan

**Keywords:** Adverse effects, Antimicrobial therapy, Risk factors

## Abstract

Linezolid has been reported to be associated with thrombocytopenia. However, limited information is available on susceptibility to thrombocytopenia after linezolid usage. We aimed to investigate the risk factors for linezolid-associated thrombocytopenia (LAT). We conducted a retrospective cohort study of patients aged ≥ 18 years who received linezolid for ≥ 5 d during hospitalization in 2019. Information was extracted from electronic medical records. Thrombocytopenia was defined as a platelet count of < 100 × 10^9^/L or a reduction from baseline ≥ 25%. Binary logistic regression and survival analyses were used to evaluate the risk factors for LAT. A total of 98 patients were enrolled. Thrombocytopenia occurred in 53.1% patients, with a median of 9 d after initiation of linezolid. There was no significant difference in the mortality or proportion of platelet transfusions between patients with and without thrombocytopenia. A higher risk of LAT was found in patients who received renal replacement therapy (RRT) (OR 4.8 [1.4–16.4]), or concurrent fluconazole (OR 3.5 [1.2–9.8]). Patients who received RRT (8 vs. 15 d) or concurrent fluconazole (11 vs. 15 d) had a shorter median time to develop thrombocytopenia. Those who simultaneously received RRT and fluconazole had the shortest median of time (6.5 d) and the highest risk of developing thrombocytopenia (87.5%).

## Introduction

Linezolid, a synthetic oxazolidinone antibiotic, has a fair activity against gram-positive pathogens. Because of its good tissue penetration, linezolid is a key therapeutic choice for nosocomial lung infections and skin or skin structure infections^1^. An oral bioavailability of 100% makes it easy to adjust for administration. According to the manufacturer’s recommendation^2^, there is no need to adjust the dose for patients with renal impairment or mild-to-moderate hepatic impairment.

Regarding the safety of linezolid, a variety of adverse effects, such as myelosuppression, peripheral and optic neuropathy, serotonin syndrome, and lactic acidosis have been reported^2^. In comparator-controlled phase III studies^3^, diarrhea, nausea, and headaches were frequent adverse effects. Although under longer treatment durations, linezolid-treated patients had a higher potential for lower platelet counts compared to the comparator, the difference was not statistically significant. However, emerging observational studies have demonstrated the thrombocytopenic effect of linezolid^4–13^. Depending on different definitions of thrombocytopenia, the incidence of thrombocytopenia ranged from 16.7% to 48.4% in adults treated with intravenous or oral linezolid^4–13^.

Although several studies have highlighted the susceptible population of linezolid-associated thrombocytopenia, the results are inconclusive. Possible risk factors including high daily dose per kg^4,6^, long duration^5,8,11,13^, renal impairment^5–8,10–13^, and low baseline platelet counts^9,11,12^ have been reported. One study^9^ showed that carbapenem combination therapy and low baseline platelet count were significant predictors, and the duration of linezolid administration or renal function had little effect. However, a study conducted by Choi et al.^11^ revealed that low-dose aspirin was a possible risk factor, and the effect of carbapenem combination therapy was not significant.

More than 60% of patients admitted to the intensive care unit may suffer thrombocytopenia^14–16^. Multiple factors predispose an individual to platelet deficiency, including sepsis, major trauma, and drug exposure^14–16^. Medications that may induce thrombocytopenia are as follows: abciximab^15^, amiodarone^11^, aspirin^11,15^, clopidogrel^15^, digoxin^11^, dipyridamole^15^, eptifibatide^15^, fluconazole^17^, haloperidol^11^, heparin^11,14–16^, oxaliplatin^16^, piperacillin^11,16^, rifampin^11,16^, ticlopidine^15^, trimethoprim-sulfamethoxazole^11,15,16^, valproic acid^11,15^, and vancomycin^15,16^.

In addition to linezolid, a variety of predisposing factors and drugs may contribute to thrombocytopenia. For clinicians, it is urgent to define a susceptible population to avoid harmful outcomes. Therefore, we conducted an observational study to evaluate the risk factors of linezolid-associated thrombocytopenia. However, whether a patient’s predisposing factors or combination treatment with certain drugs aggregates linezolid-related thrombocytopenia is still inconclusive.

## Methods

### Study design and population

We designed a retrospective cohort study to investigate the possible risk factors for linezolid-associated thrombocytopenia. Patients ≥ 18 years and hospitalized at the E-Da hospital from January 1, 2019 to December 31, 2019, were recruited into this study. We reviewed the electronic medical records of patients who received linezolid orally or intravenously for ≥ 5 d. All linezolid prescriptions were approved by the infection specialist. Patients with any of the following were excluded: A linezolid treatment duration < 5 d, baseline platelet count < 100 × 10^9^/L, received platelet transfusions within 10 d before the linezolid treatment, platelet count not monitored after starting therapy, had undergone chemotherapy or radiation for malignancy, presence of hematological diseases. Our study adhered to ethical restrictions and was approved by the Institutional Review Board of E-Da Hospital (EMRP-109–142). This observational study was performed by reviewing the medical records and the informed consent was waived by the Institutional Review Board of E-Da Hospital due to analysis of de-identified secondary data and posed no more than minimal risk of harm to study subjects.

### Individual characteristics

Individual characteristics and information from hospitalization, including age, sex, body weight, height, comorbidity, history of renal replacement therapy (RRT), date of admission, and date of discharge were collected from the medical information system. Diseases that had been documented in medical records more than three times in 6 months prior to the initiation until the end of therapy were defined as comorbidities. In addition, admission to the intensive care unit during hospitalization, and history of receiving chemotherapy or radiotherapy in the six months prior to initiation until discontinuation of linezolid were also collected.

Information about linezolid usage such as type of infection, route of administration, dosage, and dosing interval were collected from electronic medical records. Previously prescribed glycopeptides, concurrent antibiotics, and combination medications that might affect platelet count and the date of platelet transfusions were reviewed. Combination medications were defined as medications that were co-administrated with linezolid for ≥ 3 d.

### Laboratory data

Laboratory data, such as microbiological isolates, platelet count, and serum creatinine, were collected from electronic medical records.

The baseline platelet count was defined as the latest platelet count prior to linezolid treatment. The percentage change in platelet count was calculated by baseline and the lowest platelet count after initiating therapy. According to the latest version of the Common Terminology Criteria for Adverse Events (CTCAE), version 5^18^, thrombocytopenia was defined as a platelet count < 100 × 10^9^/L^15^ or a reduction from baseline ≥ 25%. Platelet counts that recovered to ≥ 150 × 10^9^/L or baseline, and platelet counts after linezolid discontinuation ≥ 14 d were also collected.

The latest data on serum creatinine levels before initiation of linezolid were collected. Baseline renal function was estimated using the Cockcroft-Gault Equation^19^. Based on the recommendation from Kidney Disease Improving Outcomes (KDIGO)^20^, the glomerular filtration rate (GFR) ≥ 90, 60–89, 45–59, 30–44, 15–29, and < 15 mL/min/1.73 m^2^ are indicated to stage 1, 2, 3a, 3b, 4, and stage 5, respectively. We divided the kidney functions into 4 categories in reference to the guideline of KDIGO^20^: creatinine clearance (CrCL) ≥ 60 (normal to mild decrease) , 30 ≤ CrCL < 60 (moderate decrease), CrCL < 30 mL/min (severe decrease), and RRT.

### Statistical analysis

Continuous and categorical variables were analyzed using the *t*-test and chi-square test, respectively. For nonparametric variables, the Mann–Whitney U test or Fisher’s exact test was used instead. The association between risk factors and thrombocytopenia was evaluated using binary logistic regression and adjustment for potential confounders. Variables with a *P*-value < 0.1, in the univariable model were further evaluated in the multivariable model. Analyses were presented as estimates and 95% confidence intervals, and *P*-values < 0.05, were considered significant. The time between initiating the treatment and the occurrence of thrombocytopenia was analyzed using Kaplan–Meier curves and was tested using the log-rank test. Statistical analyses were performed using SAS version 9.4.

### Ethics approval and consent to participate

This study adhered to ethical restriction and was approved by Institutional Review Board of E-Da hospital. Approval number was EMRP-109–142.This observational study was performed by reviewing the medical records and the informed consent was waived by the Institutional Review Board of E-Da Hospital due to analysis of de-identified secondary data and posed no more than minimal risk of harm to study subjects.

## Results

**Figure 1:**
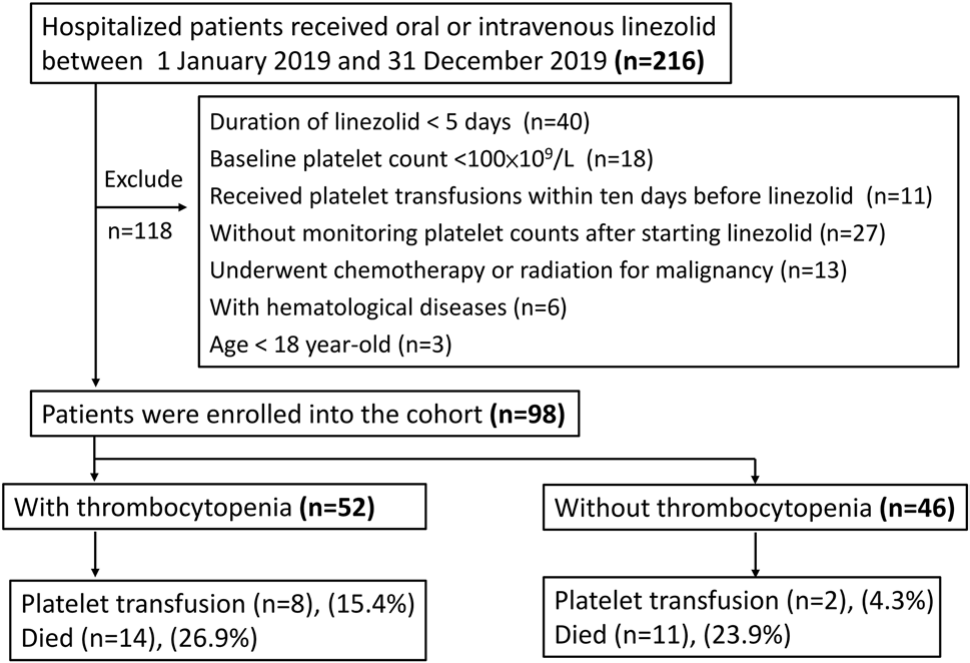
Flow chart showing enrollment into the study and clinical outcomes.

A total of 216 patients were prescribed intravenous or oral linezolid in 2019. After excluding 118 patients, 98 patients were finally included in this study (Fig. 1). Table 1 shows the individual characteristics of the 98 study subjects stratified by thrombocytopenia. The mean age of all study subjects was 69.0 ± 15.7 years and was equal in both sexes. Sixty-five patients (66.3%) received intravenous linezolid. The average treatment duration of linezolid was 13.9 ± 7.2 d. Urinary tract infection was observed in 48.0% of patients, and 52.0% of patients had a history of intensive care unit admission. Vancomycin-resistant Enterococcus isolates and oxacillin-resistant Staphylococcus isolates were present in 71.7% and 13.0% of patients, respectively.

**Table 1:** Individual and characteristics data of enrolled patients.

Characteristics	With thrombocytopenia (n = 52)	Without thrombocytopenia (n = 46)	*P*-value
Age (year), mean ± SD	71.0 ± 16.1	66.7 ± 15.2	0.1824
Male, no (%)	31 (59.62)	18 (39.13)	0.0430
BMI (kg/m^2^), mean ± SD	24.9 ± 4.4	25.0 ± 6.3	0.9529
**Renal function (mL/min), no (%)**			
CrCL ≥ 60	12 (23.08)	18 (39.13)	0.0277
30 ≤ CrCL < 60	11 (21.15)	12 (26.09)	
CrCL < 30	10 (19.23)	11 (23.91)	
Renal replacement therapy, no (%)	19 (36.54)	5 (10.87)	
Intensive care unit admission, no (%)	30 (57.69)	21 (45.65)	0.2338
**Comorbidity, no (%)**			
Hypertension	36 (69.23)	30 (65.22)	0.6724
Diabetes mellitus	29 (55.77)	14 (30.43)	0.0117
Chronic kidney disease	20 (38.46)	7 (15.22)	0.0102
Liver diseases	9 (17.31)	6 (13.04)	0.5585
**Platelet (10**^**9**^**/L), mean ± SD**			
Baseline	283.8 ± 136.1	270.7 ± 101.3	0.5870
Nadir during therapy	131.3 ± 82.9	290.7 ± 111.7	< .0001
After discontinuation (≥ 14 d)	280.5 ± 148.9	300.0 ± 122.6	0.5491
**Previous glycopeptide, no (%)**			0.4908
None	24 (46.15)	24 (52.17)	
Vancomycin	5 (9.62)	7 (15.22)	
Teicoplanin	22 (42.31)	13 (28.26)	
Both vancomycin and teicoplanin	1 (1.92)	2 ( 4.35)	
Median duration of linezolid (day)	14.0	10.5	0.0310
**Route of linezolid, no (%)**			
Oral	7 (13.46)	16 (34.78)	0.0456
Intravenous	39 (75.00)	26 (56.52)	
IV and oral	6 (11.54)	4 (8.70)	
**Types of infections, no (%)**			
Urinary tract infection	25 (48.08)	22 (47.83)	0.4087
Skin and soft tissue infection	9 (17.31)	5 (10.87)	
Bacteremia	8 (15.38)	7 (15.22)	
Intra-abdominal infection	6(11.54)	4 (8.70)	
Respiratory infection	1 (1.92)	6 (13.04)	
Others	3 (5.77)	2 (4.35)	
**Microbiological isolate, no (%)**			
Enterococcus sp.	6 (11.54)	3 (6.52)	0.3857
VRE strain	37 (71.15)	29 (60.00)	
Oxacillin-resistant Staphylococcus	4 (7.69)	8 (17.39)	
Others	5 (9.62)	6 (13.04)	
**Concurrent medications, no (%)**			
Aspirin or clopidogrel	9 (17.31)	7 (15.22)	0.7799
Amiodarone	2 (3.85)	5 (10.87)	0.2478
Piperacillin/tazobactam	5 (9.62)	4 (8.70)	0.2695
Cephalosporin	15 (28.85)	10 (21.74)	0.4205
Carbapenem	27 (51.92)	16 (34.78)	0.0879
Colistin	10 (19.23)	9 (19.57)	0.9667
Fluconazole	24 (46.15)	12 (26.09)	0.0397
Fluoroquinolone	16 (30.77)	13 (28.26)	0.7860

BMI, body mass index; CrCL, creatinine clearance; IV, intravenous; VRE, vancomycin-resistant enterococcus; SD, standard deviation.

Thrombocytopenia occurred in 52 patients (53.1%), with a median of 9 d after initiation of linezolid, and 8 of them (15.4%) received platelet transfusions. After discontinuation, the median duration of platelet counts returned to normal range was 10 d.

There was no significant difference in mortality (26.9% and 23.9%, *P* = 0.7330) and proportion of platelet transfusions (15.4% and 4.4%, *P* = 0.0716) between those with and without thrombocytopenia. Patients who developed thrombocytopenia had a significantly higher proportion of patients receiving RRT (36.5% vs. 10.9%), intravenous administration of linezolid (75.0% vs. 56.5%), male (59.6% vs. 39.1%), and longer median duration (14.0 vs. 10.5 d). In addition, a higher ratio of diabetes mellitus, chronic kidney disease, and concurrent use of fluconazole were found in thrombocytopenia (Table 1).

Table 2 shows the odds ratios of the risk factors associated with linezolid-associated thrombocytopenia. In the crude model, patients who were male, diabetic, receiving RRT, intravenous linezolid, or concurrent fluconazole therapy were found to have increased odds ratios of linezolid-associated thrombocytopenia compared with their counterparts. In the main model, we found increased odds ratios of linezolid-associated thrombocytopenia in patients who received RRT, and concurrent fluconazole therapy, with an OR of 4.773 (95% CI 1.390–16.389), and OR 3.474 (95% CI 1.230–9.810), respectively.

**Table 2:** Association between risk factors and incidence of linezolid-associated thrombocytopenia.

Variables	Crude model			Main model		
	OR	95% CI	*P*-value	OR	95% CI	*P*-value
Age	1.018	0.992–1.045	0.1851	–	–	–
Male	2.296	1.021–5.166	0.0445	2.210	0.829–5.890	0.1127
BMI	0.998	0.926–1.075	0.9512	–	–	–
Linezolid treatment duration	1.057	0.992–1.125	0.0861	1.071	1.000–1.146	0.0506
Routes of linezolid						
IV	3.427	1.239–9.480	0.0176	2.950	0.884–9.847	0.0786
Both IV and oral	3.427	0.731–16.080	0.1183	1.314	0.187–9.236	0.7840
Baseline platelet count	1.001	0.998–1.004	0.5900	–	–	–
ICU admission	1.623	0.730–3.611	0.2349	–	–	–
Renal replacement therapy	4.721	1.593–13.993	0.0051	4.773	1.390–16.389	0.0130
Hypertension	1.200	0.515–2.795	0.6726	-	-	-
Diabetes mellitus	2.882	1.253–6.628	0.0128	1.698	0.635–4.544	0.2917
Liver diseases	1.395	0.456–4.273	0.5596	–	–	–
Cephalosporin	1.459	0.580–3.671	0.4218	–	–	–
Carbapenem	2.025	0.896–4.574	0.0897	1.056	0.397–2.813	0.9125
Fluconazole	2.428	1.033–5.708	0.0419	3.474	1.230–9.810	0.0187
Fluoroquinolone	1.128	0.472–2.695	0.7865	–	–	–

Main model was adjusted for gender, treatment duration, routes of administration, renal replacement therapy, diabetes mellitus, concurrent carbapenem, and concurrent fluconazole therapy. BMI, body mass index; ICU, intensive care unit; IV, intravenous; CI, confidence interval.

**Figure. 2:**
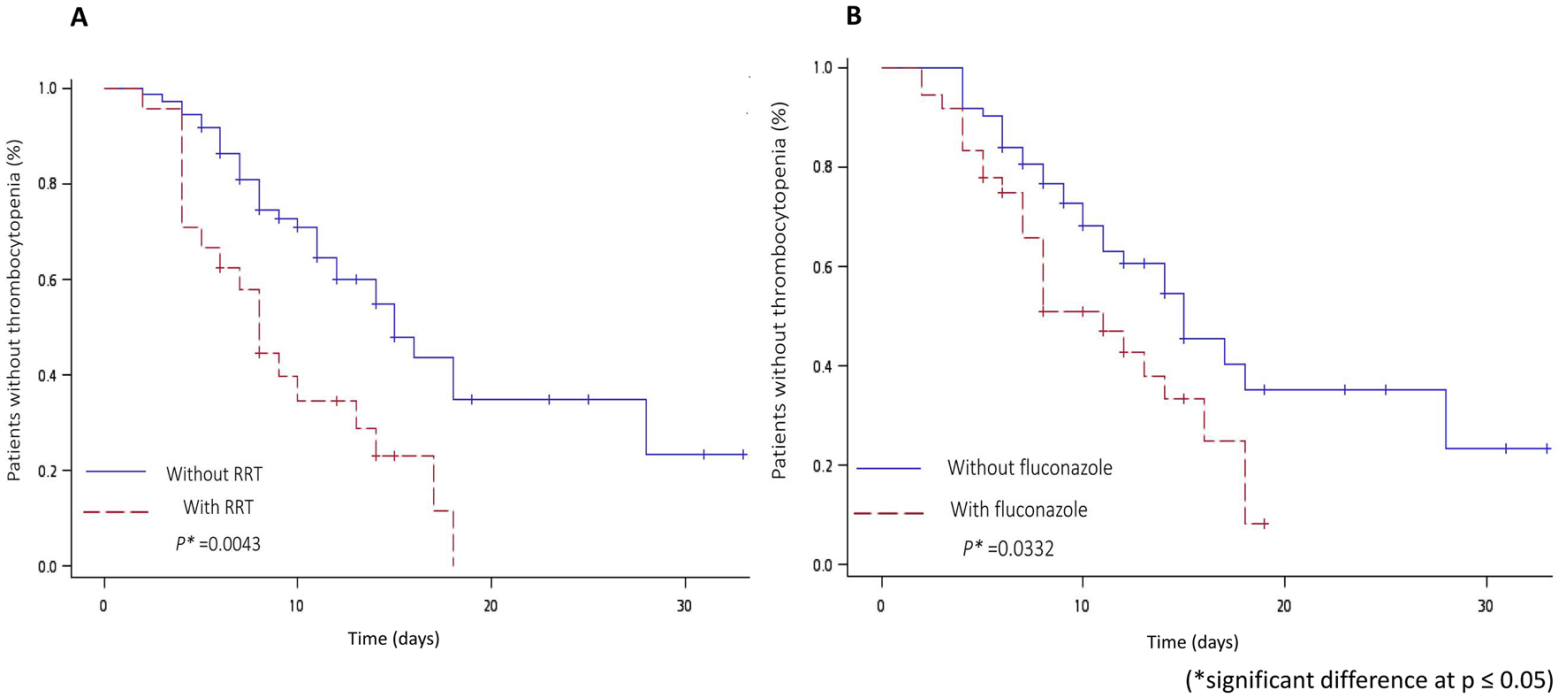
Kaplan–Meier survival curves to reveal the impact on the incidence of thrombocytopenia. (**A**) Renal replacement therapy (RRT). (**B**) Fluconazole. Difference between groups were tested by log-rank test. Patients who received RRT (log-rank test *P* = 0.043) or fluconazole treatment (log-rank test *P* = 0.0332) showed significantly higher risk of developing linezolid-associated thrombocytopenia.

Figure 2 illustrates the Kaplan–Meier survival curves of linezolid-associated thrombocytopenia events in patients who received RRT or fluconazole treatment. We found that patients who underwent RRT had a shorter median time (with vs. without RRT: 8 vs. 15 d, log-rank test *P* = 0.0043; Fig. 2) and higher risk (with vs. without RRT: 79.2% vs. 44.6%, *P* = 0.0032) of developing linezolid-associated thrombocytopenia. However, there was no significant difference in the incidence of thrombocytopenia between CrCL < 30, 30 ≤ CrCL < 60 and CrCL ≥ 60 mL/min (results detailed in Supplementary Fig. S1). Among the 24 patients who received RRT, 16 (67%) had end-stage renal disease (ESRD) who underwent RRT, while the others underwent emergent RRT for acute kidney injury (AKI). A slightly higher percentage of thrombocytopenia was noted among ESRD patients than among AKI patients, but the difference was not statistically significant (ESRD vs. AKI: 81.25% vs. 75.00%, *P* = 0.7223).

Patients who received fluconazole were also observed to have a shorter median time (with vs. without fluconazole: 11 vs. 15 d, log-rank test *P* = 0.0332; Fig. 2) and higher risk (with vs. without fluconazole: 66.7% vs. 45.2%, *P* = 0.0397) of developing linezolid-associated thrombocytopenia.

**Figure 3:**
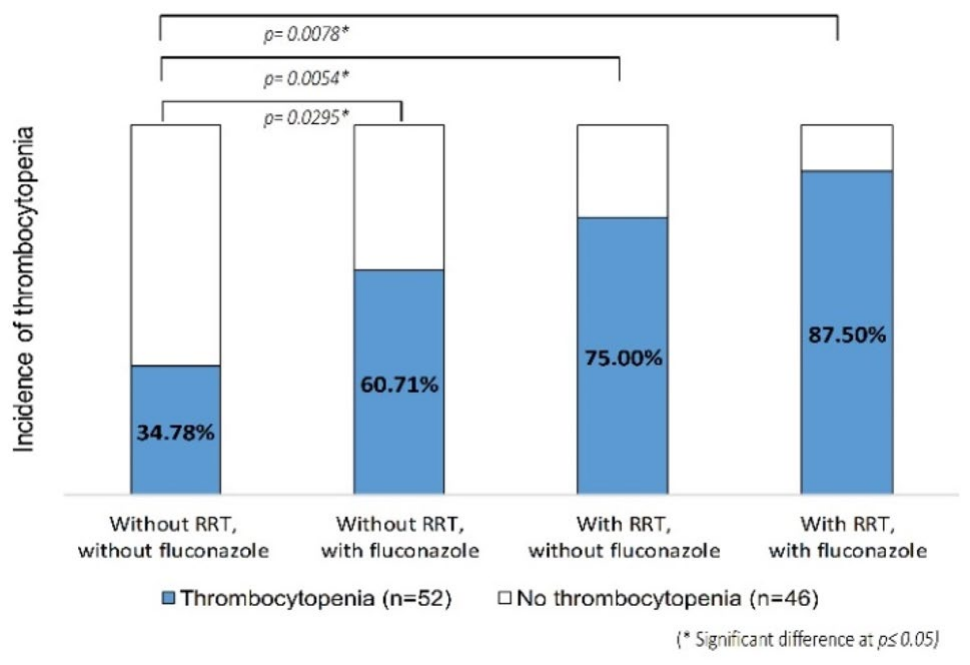
Incidence of linezolid-associated thrombocytopenia among patients who received both RRT and fluconazole, RRT alone, fluconazole alone, and denied RRT and fluconazole.

Figure 3 illustrates the subgroup analyses of linezolid-associated thrombocytopenia stratified by RRT and fluconazole therapy. The incidence of linezolid-associated thrombocytopenia was 34.8%, 60.7%, 75.0%, and 87.5%, respectively, showing an incremental trend in subgroups without RRT and fluconazole, with fluconazole, with RRT, and with both RRT and fluconazole. Multiple logistic regression showed that the odds ratios were 13.125 (95% CI 1.482–1116.267), 5.625 (95% CI 1.558–20.312), and 2.898 (95% CI 1.097–7.654), respectively, among patients who received concurrent RRT and fluconazole, RRT alone, and fluconazole alone compared with patients who did not receive RRT and fluconazole therapy.

## Discussion

To the best of our knowledge, this study is the first to demonstrate that patients who received RRT or concurrent fluconazole treatment may have a higher incidence of linezolid-associated thrombocytopenia. In addition, we revealed the real-world situation and the association between clinical risk factors and linezolid-associated thrombocytopenia.

By performing binary logistic regression, RRT and concurrent fluconazole therapy were identified as risk factors for linezolid-associated thrombocytopenia.

We discovered that patients who received RRT had a significantly higher risk (with vs. without RRT: 79.2% vs. 44.6%, *P* = 0.0032) and shorter median time (with vs. without RRT: 8 vs. 15 days, *P* = 0.0043) to have thrombocytopenia. Our finding was compatible with Hanai et al*.*’s results, which showed an onset time of 8.5 d in patients undergoing hemodialysis^8^. Hirano et al. found that patients with CrCL < 30 mL/min (60.0%) had a significantly higher incidence of thrombocytopenia then those with CrCL > 60 mL/min (26.4%)^5^. Moraza et al. and Hanai et al.^8^reported similar findings^7^. Several studies have revealed that renal function plays a critical role in thrombocytopenia associated with linezolid^5–8,10,12,21^. The manufacturer recommends that the pharmacokinetic parameters of the parent drug are not transformed^2^, and that no dosage adjustment is needed for renal impairment. However, a higher incidence of thrombocytopenia was noted among our patients who received RRT. Brier et al. concluded that the levels of two primary metabolites (PNU-142586 and PNU-142300) were higher in patients requiring hemodialysis^22^. Matsumoto et al. suggested that renal impairment elevated linezolid trough concentration, and higher drug exposure might be related to thrombocytopenia^23^. Considering the higher possibility of thrombocytopenia in renal dysfunction, physicians should be aware of the harmful outcomes, although the mechanism of linezolid-associated remains unclear.

The literature reports fluconazole-associated thrombocytopenia limited to several case reports. Pasikhova et al. reported a case of fluconazole-associated agranulocytosis with thrombocytopenia^17^. One population-based study enrolled 54,803 users of either fluconazole or itraconazole, and only 1 of 34,220 fluconazole users had thrombocytopenia^24^. So far, no interaction between fluconazole and linezolid has been found^25^. Little information is available regarding the underlying fluconazole and linezolid-associated thrombocytopenia. Further studies are necessary.

We found that patients treated with linezolid for ≥ 14 d had a relatively higher frequency of thrombocytopenia (duration ≥ 14 d vs. 14 > duration ≥ 5 d: 63.83% vs. 43.14%, *P* = 0.0403) (results detailed in Supplementary Fig. S2). Our results are compatible with those of other studies. Hirano et al ^5^ and Takahashi et al.^13^ reported a higher incidence of thrombocytopenia among patients with linezolid duration ≥ 14 d. Choi et al. ^11^ even suggested an increased odds ratio for linezolid duration ≥ 7 d. Therefore, we suggest monitoring platelet counts for prolonged durations of linezolid administration, especially for those who received RRT.

According to recent studies, the incidence of thrombocytopenia ranged from 16.7% to 48.4% in adults treated with linezolid^4–13^. Kim et al.^10^ revealed a 48.3% risk of thrombocytopenia in a cohort of 60 ICU patients, and thrombocytopenia was defined as a platelet count of < 150 × 10^9^/L or a decrease of at least 50% from the baseline. The relatively higher incidence (53.1%) in our study could have resulted from different disease severities and definitions of thrombocytopenia.

Our data showed that patients with diabetes mellitus had twice the risk of thrombocytopenia. However, this was not significant after adjusting for confounding factors. Diabetic kidney disease, a microvascular complication, frequently leads patients to undergo dialysis or renal transplantation^26^. Consequently, a higher proportion of diabetes mellitus in the thrombocytopenic group is anticipated. Regarding the administration route, IV-form linezolid was used in hospitalized patients, while oral-form linezolid was mostly prescribed for discharge in our study. Therefore, the lower risk of thrombocytopenia for oral-from linezolid in comparison to IV-from linezolid may reflect the different disease severity between discharged and hospitalized patients. Our results were compatible with the findings of Takahashi et al.^13^.

However, the mechanism underlying linezolid-associated thrombocytopenia remains unclear. Several mechanisms have been proposed. For instance, immune-mediated thrombocytopenia has been proposed by Bernstein et al*.*^27^. Other theories, including the suppression of platelets release from mature megakaryocytes^28^, and Tsuji et al. assumed that the inhibition of the platelet formation was the most common mechanism^29^. Many studies reported that the linezolid-associated adverse reactions were related to linezolid trough concentration (C_min_)^30,31^. Cojutti et al.^32^ monitored patients’ C_min_ for dose adjustment at day 3–5 of therapy, and 2–8 mg/L was taken as desired range for C_min_. Peripheral venous blood samples were collected 5 min prior to the subsequent dose. Levels of linezolid were analyzed by a validated HPLC analysis method. However, more studies are necessary to clarify the approach of dose adjustment.

Several clinical factors might also affect platelets, such as a history of surgery and recent transfusions^33^. Using radionuclides, the mean life span of transfused platelets in humans was 9.9 ± 0.6 d^34–37^. Therefore, we excluded patients who received platelet transfusions ≤ 10 d before initiation of linezolid. Moreover, the material of the dialyzer may cause thrombocytopenia; polysulfone membranes and amounts of polyvinylpyrrolidone are thought to be related to its occurrence, but the exact mechanism remains to be elucidated^38^.

Our observational cohort study has several limitations. First, due to the retrospective nature of the study design, we could not make causal inferences. Second, means of monitoring concentrations of linezolid and its metabolites were not available, and it was difficult to evaluate the pharmacokinetic parameters of linezolid in thrombocytopenic patients. Monitoring linezolid C_min_ at steady-state condition may be a feasible approach. However, therapeutic drug monitoring of linezolid is in experimental stage, and more studies are warranted. Third, linezolid is primarily metabolized in liver^39^, but the value of liver enzymes including aspartate aminotransferase or alanine aminotransferase were not routinely checked for all patients. We used underlying liver disease as a surrogate in the statistic to examine the potential influence of liver function on the relationship of linezolid-related thrombocytopenia. The estimate of liver disease was statistically insignificant in the crude model of logistic regression, and the covariate of liver disease was not further selected in the multivariable model. Fourth, the small sample size of this study may result in the wide ranges of confidence intervals and insignificant statistics for some interested covariates, and the interpretation of our results had to be cautiously.

## Conclusions

Renal adjustment is not required for linezolid according to the official recommendation. However, in our study, we found that RRT, and concurrent fluconazole were significantly associated with the risk of thrombocytopenia. Patients who received RRT had a significantly shorter median time to thrombocytopenia after initiation of linezolid. Careful monitoring of platelets is warranted for patients with renal impairment, especially for those already receiving RRT. However, due to the retrospective nature of our study, large randomized control trials are warranted to verify the association between possible clinical factors and the risk of linezolid-associated thrombocytopenia.

## Supplementary Information

Supplementary Information.

## Data Availability

The datasets analyzed during the current study are available from the corresponding author on reasonable request.

## Abbreviations

AKI: Acute kidney injury
BMI: Body mass index
CI: Confidence interval
CKD: Chronic kidney disease
CrCL: Creatinine clearance
CTCAE: Common Terminology Criteria for Adverse Events
ESRD: End stage renal disease
ICU: Intensive care unit
IV: Intravenous
OR: Odds ratio
LAT: Linezolid-associated thrombocytopenia
RRT: Renal replacement therapy
SD: Standard deviation
VRE: Vancomycin-resistant enterococcus
