# Supplementary material for: Renal replacement therapy and concurrent fluconazole therapy increase linezolid-related thrombocytopenia among adult patients
Source: Sci Rep. 2022 Jun 14;12:9894. doi: 10.1038/s41598-022-13874-y (PMC9198091; doi:10.1038/s41598-022-13874-y)
Supplement: Supplementary file 1 — Supplementary Information. [file 41598_2022_13874_MOESM1_ESM.docx]

Supplementary figures: Incidence of thrombocytopenia stratified by renal function or duration of linezolid

Supplement figure S1


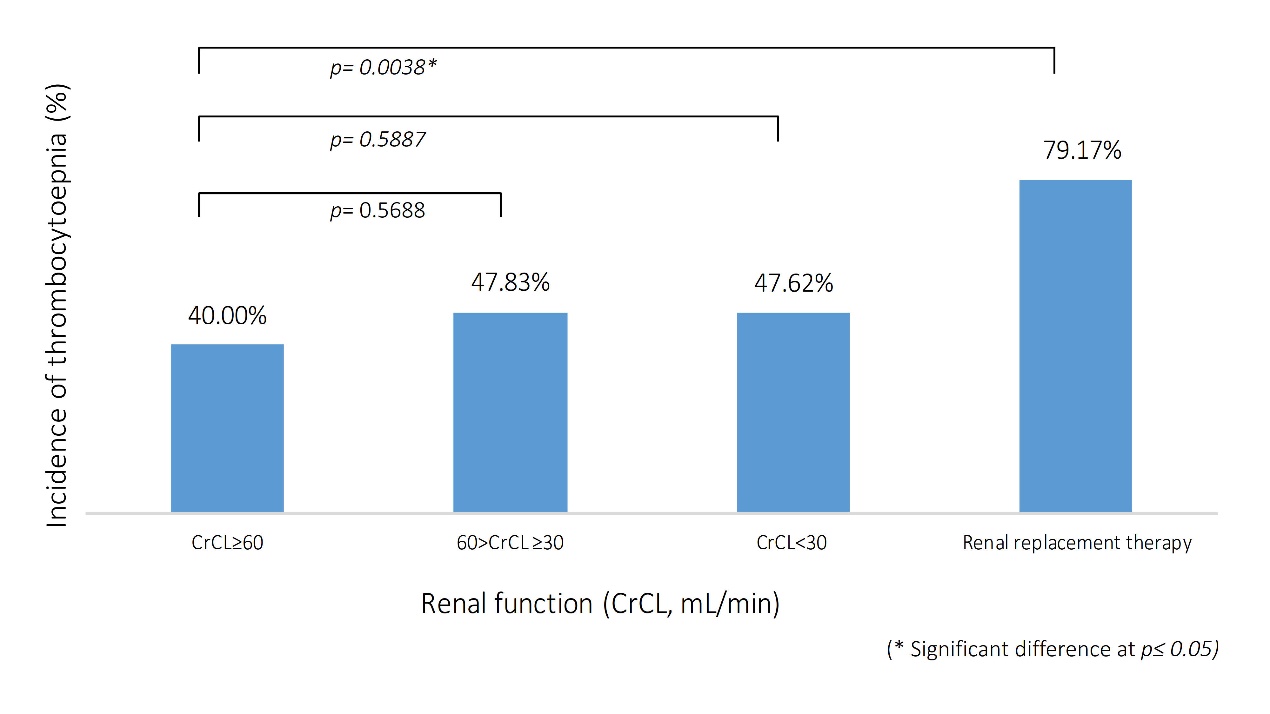


Figure S1.

Enrolled patients were classified into four groups according to renal function, including creatinine clearance (CrCL)≥ 60, 60> CrCL≥ 30, CrCL< 30 mL/min, and renal replacement therapy (RRT). Patients received RRT were with higher risk to develop thrombocytopenia (with vs without RRT: 79.2% vs 44.6%, *P*=0.0032). But, there was no significant difference between CrCL≥ 60, 60 >CrCL≥ 30, CrCL< 30 mL/min.

Supplementary figures: Incidence of thrombocytopenia stratified by renal function or duration of linezolid

Supplement figure S2


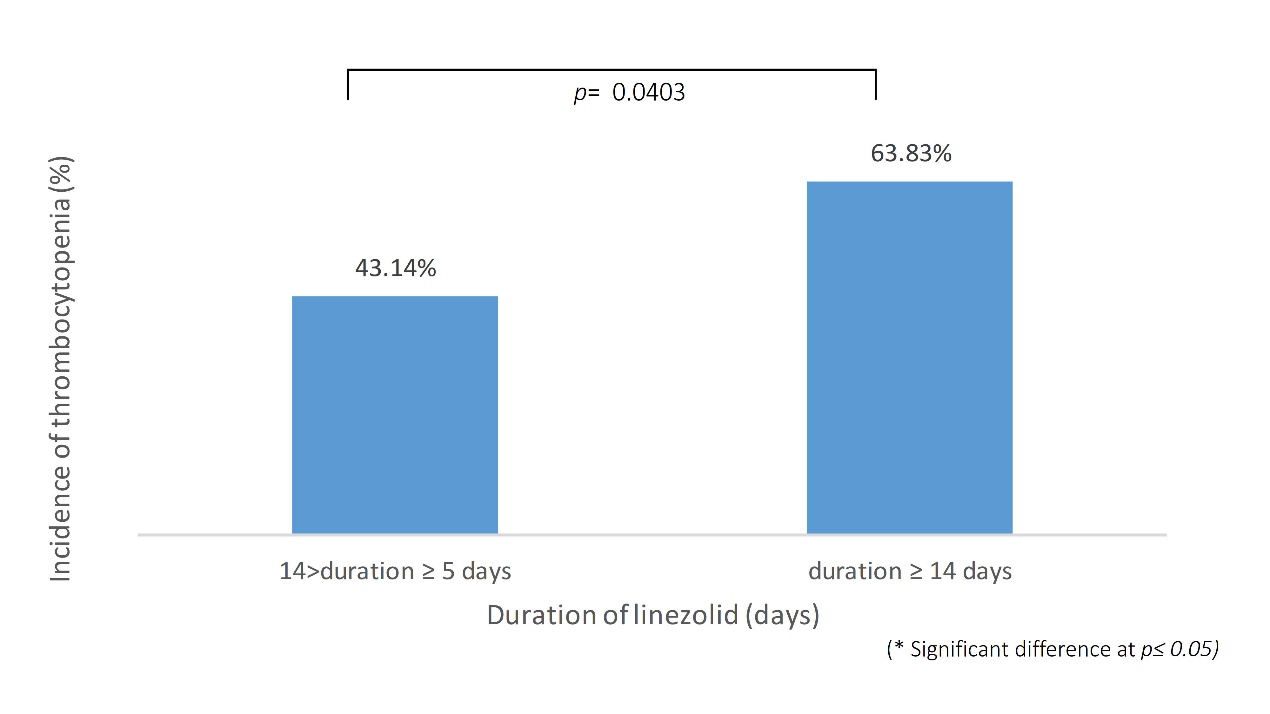


Figure S2.

All patients were also classified into two groups according to the duration of linezolid, including 14>duration≥ 5 and duration≥ 14 days. Patients with duration≥ 14 days were with higher risk of thrombocytopenia (14>duration≥ 5 days vs duration≥ 14 days: 43.14% vs 63.83% *P* = 0.0403).
